# Role of Multidetector Computed Tomography with Multiplanar and Curved Multiplanar Reformations in the Detection of Cause of Intestinal Obstruction: A Tertiary Care Experience

**DOI:** 10.7759/cureus.7464

**Published:** 2020-03-29

**Authors:** Anum Sultan, Maria Hassan, Muhammad Ali

**Affiliations:** 1 Radiology, Dr. Ziauddin Hospital, Karachi, PAK

**Keywords:** intestinal obstruction, mdct

## Abstract

Objective

To determine the role of multidetector computed tomography (MDCT) with multiplanar (MPR) and curved multiplanar reformations (CMPR) in the detection of the cause of intestinal obstruction.

Materials and methods

A retrospective analysis of 200 patients with a clinical suspicion of intestinal obstruction referred to the department of radiology, Dr. Ziauddin University Hospital, Clifton campus, from September 2016 to October 2019, was done. All patients who underwent an MDCT scan with oral and intravenous (I/V) contrast were included in the study. Patients with deranged serum creatinine and an allergic reaction to contrast were excluded from the study. MPR and CMPR images were acquired in each patient in addition to routine axial images. The causes of intestinal obstruction as determined by a computed tomography (CT) scan were confirmed on surgery and colonoscopy. The CT scans were analyzed by an independent radiologist with five years of experience blinded to the surgical and colonoscopy findings in detecting the cause of bowel obstruction using the axial, MPR, and CMPR images. Data analysis was done on IBM SPSS Statistics for Windows, Version 20.0 (IBM Corp., Armonk, NY).

Results

Out of 200 patients with a clinical suspicion of intestinal obstruction, 120 patients with intestinal obstruction was confirmed on CT scan. Fifty-eight patients were males (48.33%) and 62 patients were females (51.66%) with a male-to-female ratio of 1:1.06. The mean age of the patients was 54.7 years (age range from 06 years to 85 years). Abdominal distension was the most common presentation seen in 37 patients (30.83%) followed by vomiting in 25 patients (20.83%). Small bowel obstruction was seen in 96 patients (80.00%) with the ileum being the most common site of obstruction seen in 76 patients (63.33%). Among the patients with the ileum being the site of obstruction, distal ileal obstruction was seen in the majority of patients (30 patients, with a frequency of 25.00%). Twenty-three patients (19.16%) had a large bowel obstruction, with sigmoid colon involvement seen as the most common site in 10 patients (8.33%). Adhesions were the leading extrinsic cause of bowel obstruction seen in 32 patients (26.6%). Intraluminal causes of obstruction were seen in 36 patients (30.0%) with carcinoma being the commonest cause (12 patients with a frequency of 10.0%). A foreign body is the primary cause of intraluminal obstruction (three patients, with a frequency of 2.5%). The sensitivity, specificity, positive predictive value, and negative predictive value of MDCT were 86.2%, 92.7%, 90.1%, and 96.4%, respectively.

Conclusion

MDCT has high sensitivity and specificity to diagnose and determine the cause of bowel obstruction. It not only determines the site of obstruction but also the cause of obstruction, including intrinsic, extrinsic, and intraluminal causes.

## Introduction

Intestinal obstructions account for 20% of hospital admissions for acute abdomen requiring surgical consultation [1]. It has been observed that the small bowel is more frequently involved, accounting for 60%-85% of cases of intestinal obstruction with a four to five times less common involvement of the large bowel [1-2]. It occurs either due to mechanical obstruction or functional abnormality that results in the interruption of the normal passage of intraluminal contents [3]. Various causes of bowel obstruction have been identified, which can be categorized as extrinsic, intrinsic, or intraluminal based on their location [4]. Extrinsic causes include adhesions, volvulus, hernia (leading to a closed-loop obstruction or strangulation) and intra-abdominal masses (neoplasms, diverticulitis, appendicitis). Intrinsic causes are neoplasms, intussusception, intramural hematoma, and inflammatory and infective conditions (Crohn’s disease and tuberculosis) [4-5]. Polyps, ingested foreign bodies, and bezoars are the common intraluminal causes of bowel obstruction [5]. Small bowel obstruction is most commonly caused by extrinsic lesions such as adhesions and hernia. Conversely, intrinsic lesions, such as neoplasm or inflammatory condition, contribute mostly to large bowel obstruction [4-5].

It is of immense importance to promptly diagnose the cause of bowel obstruction to prevent severe complications such as ischemia and bowel necrosis [1,6]. The diagnosis of bowel obstruction is made on the basis of history, clinical examination, and radiological findings [7]. Radiological investigations include plain radiographs, contrast studies, and advance imaging such as computed tomography (CT) scans [8]. Plain radiographs have low sensitivity, specificity, and accuracy of 69%, 57%, and 46%-80%, respectively, in determining the presence of bowel obstruction with even lower accuracy in determining the cause and site of obstruction [5,8]. A CT scan is a widely used imaging modality for the diagnosis of intestinal obstruction and can detect the cause of obstruction in 93% to 95% of cases. Depending on the grade of obstruction, the overall reported sensitivity, specificity, and accuracy of CT scans is 63%, 78%, and 66%, respectively [4,9].

The purpose of this study is to determine the causes and level of small and large bowel obstruction using MDCT and its post-processing techniques, including multiplanar reformations (MPR) and curved multiplanar reformations (CMPR), enhancing the initial diagnosis, which, in turn, helps the surgeon in surgical planning, thus saving the patient from adverse complications.

## Materials and methods

A retrospective analysis of a database of 200 patients with the clinical suspicion of intestinal obstruction referred to the department of radiology, Dr. Ziauddin University Hospital, Clifton campus, from September 2016 to October 2019, was done. As the study was retrospective in nature, informed consent was waived. The patient’s records were evaluated for presenting symptoms and imaging findings to assess the cause and site of intestinal obstruction. Surgical and medical records were reviewed to confirm the CT findings. The clinical suspicion of bowel obstruction was based on the presence of symptoms such as abdominal distension, abdominal pain, vomiting, nausea, constipation, abdominal tenderness, or abnormal bowel sounds.

All patients who underwent an MDCT scan with oral and intravenous (I/V) contrast before surgical exploration and colonoscopy were included in the study. All examinations were performed on multidetector Asteion™ 16 (Toshiba, Japan). The CT protocol included scanning in a single breath-hold from the dome of the diaphragm to the symphysis pubis. Fifteen ml of gastrografin (Gastrografin, Bayer, Berlimed SA, Spain) diluted in 1.5 liters of water was used as oral contrast. The administration of oral contrast was commenced four hours prior to the CT examination. Five-hundred ml of oral contrast was administered immediately before CT image acquisition. I/V non-ionic contrast iopromide (Ultravist 370, Bayer Pharma AG, Germany) was injected according to the body weight (1.5 ml/kg) through an 18-gauge cannula at the rate of 3 ml/sec using an automated power injector. Imaging was done in the arterial, portovenous, and delayed phases, taken at 15-20 seconds, 40-60 seconds, and five minutes, respectively, with a slice thickness of 5 mm, collimation of 2 mm, pitch of 1.35, at 120kvp, 250mAs, and medium field of view. MPR with CMPR were acquired in each patient along with routine axial images. Data analysis was done on IBM SPSS Statistics for Windows, Version 20.0 (IBM Corp., Armonk, NY). The patients with deranged serum creatinine and allergic reaction to contrast were excluded from the study.

CT scans were analyzed by a radiologist having five years of experience and blinded to surgical and colonoscopy findings for the detection of the cause and site of bowel obstruction using the axial, MPR, and CMPR images. The causes of obstruction were categorized as extrinsic, intrinsic, and intraluminal. Lesions were labeled as extrinsic when their epicenter is outside the bowel, causing mass effect and resulting in bowel obstruction. Pathologies inherent to the bowel, either infective, inflammatory, or others causing bowel obstruction, were considered intrinsic causes. Bowel obstructions by masses within the bowel lumen with the subsequent obliteration of the bowel lumen were included as intraluminal causes. The site of bowel obstruction was also evaluated.

## Results

Out of 200 patients with a clinical suspicion of intestinal obstruction, 120 patients with an intestinal obstruction were confirmed on CT scan. Fifty-eight patients were males (48.33%) and 62 patients were females (51.66%) with a male-to-female ratio of 1:1.06. The mean age of the patients was 54.7 years (age range from 06 years to 85 years). Abdominal distension was the most common presentation seen in 37 patients (30.83%) followed by vomiting in 25 patients (20.83%). The presenting symptoms of patients are tabulated in Table 1.

**Table 1:** Distribution of the patients according to presenting symptoms

CLINICAL SYMPTOMS	NUMBER OF PATIENTS	FREQUENCY OF SYMPTOMS (%)
Abdominal distension	37	30.83
Vomiting	25	20.83
Abdominal pain	19	15.83
Constipation	19	15.83
Nausea	05	04.16
Others	15	12.50
Total	120	100

Small bowel obstruction was seen in 96 patients (80.00%) with the ileum being the most common site of obstruction seen in 76 patients (63.33%). Among the patients with the ileum being the site of obstruction, a distal ileal obstruction was seen in the majority of patients (30 patients) with a frequency of 25.00%. Twenty-three patients (19.16%) had large bowel obstruction with sigmoid colon involvement seen as the most common site in 10 patients (8.33%). The site of obstruction of the bowel is given in Table 2.

**Table 2:** Distribution of patients according to the site of bowel obstruction

SITE OF OBSTRUCTION	NUMBER OF PATIENTS	FREQUENCY OF BOWEL INVOLVEMENT (%)
Duodenum	03	2.5
Jejunum	17	14.1
Ileum	76	63.3
Ileocecal junction	01	0.8
Cecum	03	2.5
Ascending colon	04	3.3
Transverse colon	03	2.5
Descending colon	02	1.6
Sigmoid colon	10	8.3
Rectum	01	0.8
Total	120	100

Extrinsic causes were seen in 79 patients and account for most cases of intestinal obstruction (65.8%). Adhesions were the leading extrinsic cause of bowel obstruction seen in 32 patients (26.6%). Intraluminal causes of obstruction were seen in 36 patients (30.0%) with carcinoma being the commonest cause (12 patients with a frequency of 10.0%). A foreign body is the primary cause of intraluminal obstruction (three patients with a frequency of 2.5%).

Different causes of bowel obstruction are tabulated in Table 3.

**Table 3:** Distribution of patients according to the causes of bowel obstruction

CAUSES OF BOWEL OBSTRUCTION		NUMBER OF PATIENTS	FREQUENCY OF CAUSE OF BOWEL OBSTRUCTION (%)
Extrinsic	Adhesions	32	26.6
	Mesenteric ischemia	19	15.8
	Hernia	16	13.3
	Volvulus	06	5.0
	Abdominal collection	03	2.5
	Pregnancy	01	0.8
	Diverticulitis	01	0.8
	Appendicitis	01	0.8
Intrinsic	Carcinoma	12	10.0
	Tuberculosis	09	7.5
	Perforation	06	5.0
	Inflammatory	05	4.1
	Intussusception	03	2.5
	Meckel’s diverticulum	01	0.8
Intraluminal	Foreign body	03	2.5
	Polyp	02	1.6
Total		120	100

Statistical analysis, including sensitivity, specificity, positive predictive value, negative predictive value, and kappa of MDCT in detecting different causes of bowel obstruction were also calculated, as shown in Table 4.

**Table 4:** Sensitivity, specificity, positive predictive value, and negative predictive value of MDCT in detecting different causes of bowel obstruction MDCT: multidetector computed tomography

CAUSE OF OBSTRUCTION	SENSITIVITY (%)	SPECIFICITY (%)	POSITIVE PREDICTIVE VALUE	NEGATIVE PREDICTIVE VALUE	KAPPA
Adhesions	100	100	100	100	1
Mesenteric ischemia	100	100	100	100	1
Hernia	100	100	100	100	1
Volvulus	100	100	100	100	1
Abdominal collection	100	100	100	100	1
Pregnancy	100	100	100	100	1
Diverticulitis	7.7	84.5	25.6	89.5	0.3
Appendicitis	100	100	100	100	1
Carcinoma	100	100	100	100	1
Perforation	100	100	100	100	1
Tuberculosis	18.8	53.8	75	58.3	0.51
Inflammatory	54.2	46.2	41.6	95.8	0.54
Intussusception	100	100	100	100	1
Meckel’s diverticulum	100	100	100	100	1
Foreign body	100	100	100	100	1
Polyp	100	100	100	100	1

## Discussion

Intestinal obstruction is a frequently encountered emergency condition requiring urgent surgical management. The early diagnosis and detection of intestinal obstruction is of utmost importance to prevent bowel ischemia and necrosis and the resultant bowel resection. MDCT has been utilized as a fast and accurate technique in the detection of the cause and site of bowel obstruction as well as its complications, thus providing help to plan appropriate measures to achieve the desired outcome.

With the increased incidence of abdominal surgeries, adhesions have emerged as the most frequent cause of bowel obstruction. It is reported that 60% of small bowel obstructions are due to adhesions, with 80% of cases occurring after surgery [10]. Adhesive small bowel obstruction is seen in 3% of all laparotomies and can occur as early as within three days to two weeks, with 40% formed within one year [11]. Other causes of adhesion are inflammation, seen in 15% of cases, and rare cases are due to congenital or unexplained causes [10-11]. Pongpornsup S et al. studied the various causes of bowel obstruction and found adhesions as the most common cause of bowel obstruction. He reported that adhesions were seen in 36% of patients of small bowel obstruction found in nine out of 25 patients [12]. In a study by Memon W et al., out of 120 patients, 59 (57.8%) were found to have adhesions, with 19 patients having adhesions at multiple levels [6]. The results of our study are in concordance with the results of these studies.

In our study, one case is of a 33-year-old patient. She was primigravida with four months gestational amenorrhea. She presented with complaints of nausea, abdominal pain, and constipation. Her MDCT imaging revealed an intra-abdominal pregnancy, causing obstruction at the level of the ileum, resulting in dilatation of the proximal bowel loops. Webster PJ et al. had reviewed the database of pregnant patients resulting in bowel obstruction. He found that 60% of small bowel obstructions in pregnant patients was due to adhesions. Other causes include volvulus, internal herniation, and mesenteric bands. No case of intra-abdominal pregnancy causing a bowel obstruction has been reported so far. This was a rare case of small bowel obstruction [13].

**Figure 1:**
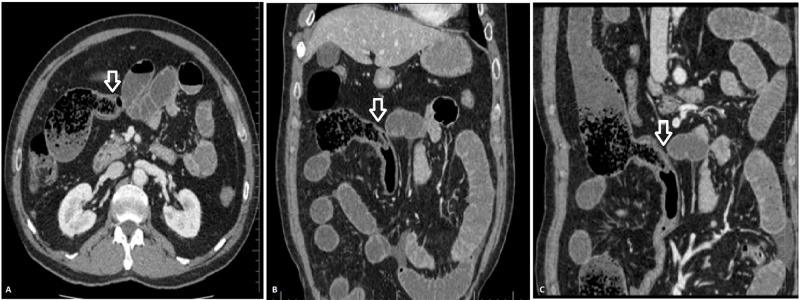
MDCT axial (A), coronal (B), and sagittal (C) images showing inverted Meckel’s diverticulum (arrow) resulting in small bowel obstruction with small bowel feces sign in the distal ileum MDCT: multidetector computed tomography

Another unusual case is that of a 62-year-old patient who presented with abdominal pain, abdominal distension, and generalized tenderness. His MDCT scan revealed small bowel feces sign in distal ileal loops. There is dilatation of the proximal small bowel loops with collapsed distal ileal loops and large bowel. On CMPR, a small segment of bowel was seen herniating into the distal ileum serving as a lead point of distal ileal obstruction. It was diagnosed as inverted Meckel's diverticulum and the diagnosis was confirmed on surgery (Figure 1). Few case reports have been published that report an inverted Meckel’s diverticulum as the cause of small bowel obstruction [14-15].

**Figure 2:**
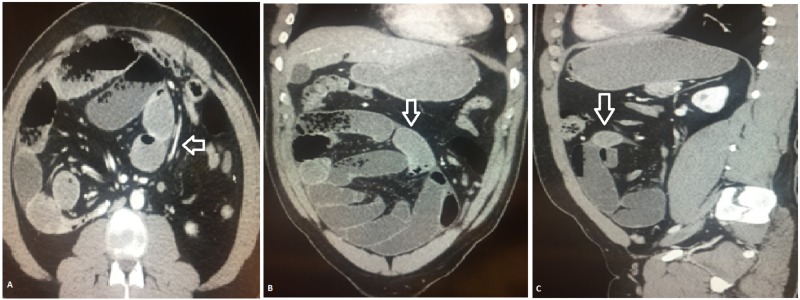
MDCT axial (A), coronal (B), and sagittal (C) images showing internal herniation of the small bowel with cocoon formation (arrow) MDCT: multidetector computed tomography

One of our patients had internal herniation on MDCT scan with the congregation of dilated small bowel loops in the right lower abdomen with mild engorgement of mesenteric vessels. There was no ascites. The diagnosis of closed-loop obstruction with abdominal cocoon formation was made (Figure 2). Tombak MC et al. have reported cases of an abdominal cocoon and found it to be a rare cause of small bowel obstruction that needs consideration in the differential diagnosis [16].

Colorectal carcinoma, volvulus, and diverticulitis account for approximately 80%-85% of all large bowel obstructions. According to the study by Jaffe T et al., malignancy is the commonest cause of large bowel obstruction seen in 60%-80% cases [17-18]. This is followed by volvulus, which accounts for 11%-15% of cases of large bowel obstruction [18]. Most cases of large bowel obstruction in our study are seen are due to carcinoma involving the cecum and ascending colon followed by volvulus. Colonic carcinoma accounts for 12 cases of large bowel obstruction. There are six cases of volvulus involving the sigmoid colon. Valsdottir E reported that volvulus accounts for 10%-13% of all large bowel obstructions with sigmoid colon involvement seen in 70%-80% of cases [19]. The results of our study were similar to these studies.

The main limitation of this study is that it is a retrospective study. The second limitation is that few patients included in the study were conservatively managed, whose diagnosis was not nearly so certain. The third limitation was that patients with renal failure could not be given intravenous contrast and hence were not included in the study.

## Conclusions

MDCT has high sensitivity and specificity to diagnose and determine the cause of bowel obstruction. It not only determines the site of obstruction but also the cause of obstruction, including intrinsic, extrinsic, and intraluminal causes.
